# Minimally invasive surgery of diabetic foot – review of current techniques

**Published:** 2016

**Authors:** I Botezatu, D Laptoiu

**Affiliations:** * Colentina Clinical Hospital, Bucharest, Romania

**Keywords:** corrective diabetic foot surgery, minimally invasive, arthrodesis, midfoot fusion bolts, headless compression screw

## Abstract

The term diabetic foot is usually used to indicate advanced foot pathology (complex clinical situations correlating diabetic foot ulcers, diabetic foot infections, Charcot foot, and critical limb ischemia). The early recognition of the etiology of these foot lesions is essential for the therapeutic decision in order to achieve a good functional result. Several surgical procedures involving the foot have been developed in order to promote healing and avoid complications. Traditionally, surgery has been performed in an open way. The literature regarding the performance and efficacy of classical osteotomies and arthrodesis is inconsistent. This can be attributed to several variables, such as differences in patient clinical aspects and the panel of surgical techniques utilized. As with other surgical specialties, fluoroscopic imaging and minimally invasive tools are now being incorporated in these procedures. The use of high speed burrs associated with specialized osteosynthesis implants, offers several advantages over classical techniques. The ability to associate these gestures to complex protocols is beginning to be currently developed. The respect for the soft tissues is considered one of the first advantages. Despite the limited time since they were introduced in clinical practice, functional results seemed to be consistent, supporting the use of this technology.

## Introduction

Of all the late complications associated with diabetes, foot pathology is the easiest to detect. The latest 20 years brought new surgical proto cols suggesting relatively simple interventions with an amputation rate reduction by 50-80% [**1**].

The term diabetic foot is usually used to indicate advanced foot pathology (complex clinical situations associating diabetic foot ulcers, diabetic foot infections, Charcot foot, and critical limb ischemia).

Ulceration and infection of the lower extremities are the leading causes of hospitalization in patients with diabetes, and treating them continues to be a medical and surgical challenge. The pathophysiology of diabetic foot disorders is multifactorial and includes neuropathy, infection, ischemia, followed by modifications in the skeletal foot structure and biomechanics [**2**]. The early recognition of the etiology of these foot lesions is essential for the therapeutical decision in order to obtain a good functional result.

Foot ulcers commonly become infected and lead to amputation. Approximately 85 percent of the patients with diabetes who suffer an amputation have foot ulcers [**3**]. Healing foot ulcers and preventing their recurrence could prevent most amputations in patients with diabetes. Foot ulcer treatment consists of a protocol involving infection management, vascular amelioration, and removal of mechanical stress areas in the foot [**4**]. As mentioned, biomechanical modifications are a compound function of neuropathy, bone deformity, and associated gait disturbances. This is an important area, where orthopedic surgeons can get involved to avoid allowing the development of areas of plantar hyper-pressure that would induce recurrence of ulceration [**5**].

The above mentioned structural deformities and gait abnormalities are sometimes secondary to the so-called intrinsic minus foot (IMF - complex foot pathology associating claw toes, pes cavus, depressed metatarsals) related to the loss of intrinsic muscle stability with long flexor domination, anterior crural atrophy (tibialis anterior, extensor hallucis longus) with weakness and foot drop, equinus deformity because of dominant triceps surae, Charcot deformity with rocker bottom aspect (Lisfranc and metatarsophalangeal joint - MPJ subluxation).

Another consequence of neuropathy is LJM (limited joint mobility), which is also due to conjunctive tissue glicosylation inducing fibrosis of the joint (especially subtalar joint), soft tissue and skin.

The atrophy of the intrinsic muscle of the foot, predominantly plantar flexors of toes, alters flexor/ extensor balance at MPJ-claw toes and prominent metatarsal heads (due to the pushing forward of the fibro-fatty metatarsal cushions).

The non-enzymatic glicosylation of collagen leads to the cross-linkage of collagen bundles, stiffness of ligaments – with limitations in the range of motions of the joint of foot particularly in subtalar joint and ankle, leading to an increased plantar pressure and the altering of the mechanism of walking. Ulcers occur at sites of high pressure on either plantar or dorsal surface [**6**]. These lead to a relatively rigid and unstable foot with altered weight bearing pressure areas.

**Fig. 1:**
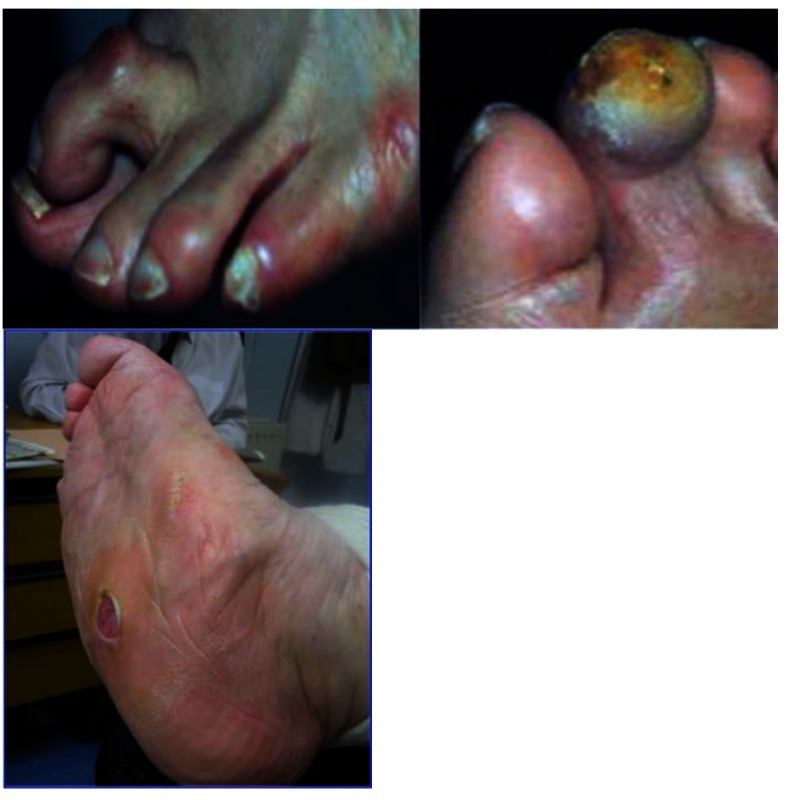
Clinical appearance of several deformities associated with the diabetic foot: claw toes on left, Charcot with plantar ulcer on right

Charcot foot is another complex progressive condition consequent to joint dislocation, pathologic fractures, and severe deformity that often signs the onset of plantar soft tissue breakdown and ulceration due to the plantar dislocation of the tarsal bones and instability of the foot. Midfoot problems are typical and the collapse of the arch creates a typical clinical aspect of the rocker-bottom deformity. The most frequent ulcer locations are plantar medial (with the dislocation of the medial cuneiform or navicular bone+/ -talus bone), followed by plantar lateral ulcers (dislocation of cuboid bone) and plantar central ulcers (collapse of the central tarsal-metatarsal region) (**Fig. 1**).

According to the International Consensus on the Diabetic Foot, published by the IWGDF in 1999 [**7**], the prevention and treatment of complications in the diabetic foot should be organized at three levels.

Level 1: involving general practitioners, nurses, and podiatrists and targeting patient awareness of foot problems and prevention, as well as an early diagnosis of ulceration.

Level 2: requiring diabetologists, specialized nurses, and surgeons (general and / or vascular and / or orthopedic) for the basic preventive and curative care.

Level 3: reference centers that should have a close multidisciplinary teamwork between the diabetologist, orthopedic surgeon, and vascular surgeon, to manage the most difficult cases: deep infected ulcer, severe arteriopathy, and Charcot foot. As it can be seen, starting from the second level, the role of surgery in promoting healing and avoiding complications can be clearly highlighted.

## Material and method

Minimally invasive surgery – MIS allows the performance of interventions through small incisions. However, MIS means not only small incisions but also less soft tissue trauma. This plays an important role for the diabetic foot given the frequency of soft tissue complications.

The initial pathology related to diabetes complications is the intrinsic minus foot, in which claw toes and metatarsal heads prominence can be easily diagnosed and surgically corrected [**8**].

**Fig. 2:**
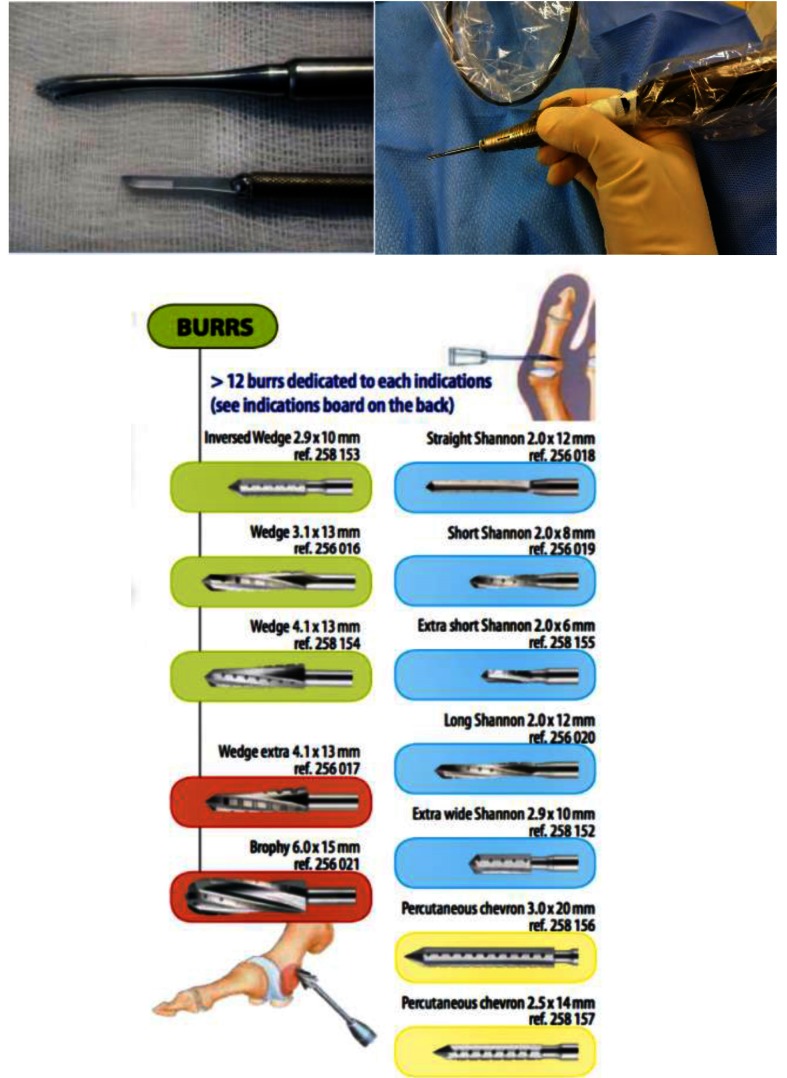
Specialized tools used in the minimally invasive surgery of the foot: rasp and beaver blade on top, motorized high-speed burr on the right (POD ®tools provided by Fournitures Hospitalieres)

The surgical off-loading can be done by percutaneous surgery, several interventions being well-developed - distal metatarsal and phalanx osteotomies, tenotomies, capsulotomies. Being minimally traumatic and not requiring osteosynthesis, it potentially decreases the risk of infection, vascular complications, and healing problems in diabetic patients. As special requirements, these techniques associate specific tools (beaver blade, motorized burrs, specific power machine – **Fig. 2**) with fluoroscopy control and an important learning curve.

**Fig. 3:**
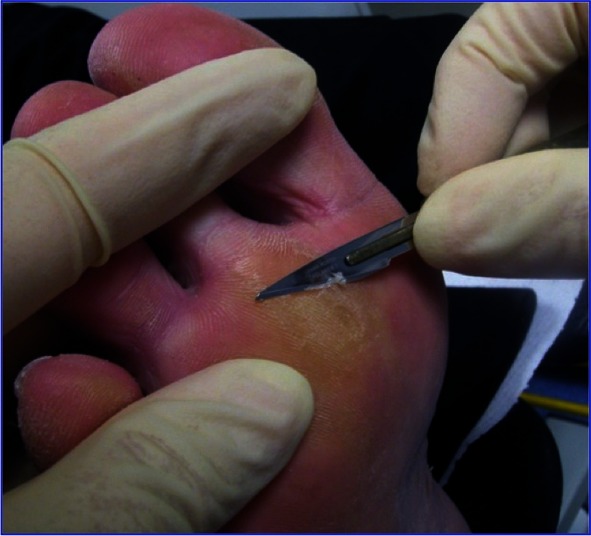
Plantar debridement for the release of an abscess

Claw toe is a deformity characterized by a hyperextension and subluxation of a metatarsophalangeal joint, with flexion deformity of the interphalangeal joints and transfer of weight bearing to the metatarsal heads. It may be due to neuropathy, muscle imbalance, or joint disease and typically affects all toes except for the first. Patients with diabetes and neuropathies can have a callus or erythema over the dorsal proximal interphalangeal - PIP joint where it touches the shoe or a callus at the tip of the toe and a malformed nail. When pain beneath the callus exceeds the neuropathic level in a patient with diabetes, an abscess may be present beneath the callus, which is discovered only if the callus is debrided (**Fig. 3**).

**Fig. 4:**
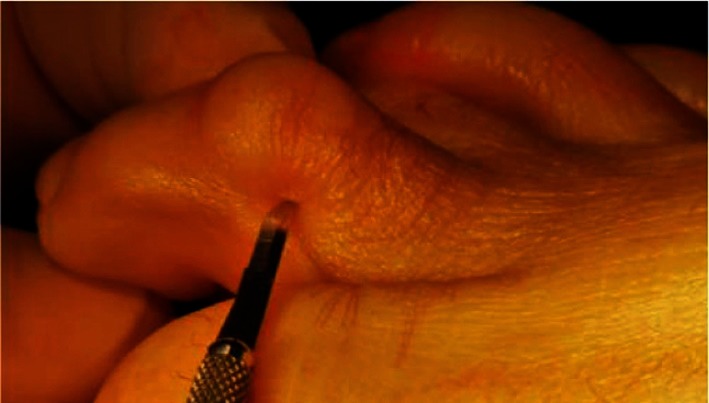
Release of the flexor tendons through lateral approach using the specialized beaver blade

For the PIP flexion, the deformity repair regarding the GRECMIP [**9**] concerns the suggested surgical protocol, which requires the association of the following: PIP plantar release (as in **Fig. 4**) + FDB tenotomy + P1 osteotomy (PIP = proximal interphalangeal joint, FDB = flexor digitorum brevis, P1 = first phalanx).

**Fig. 5:**
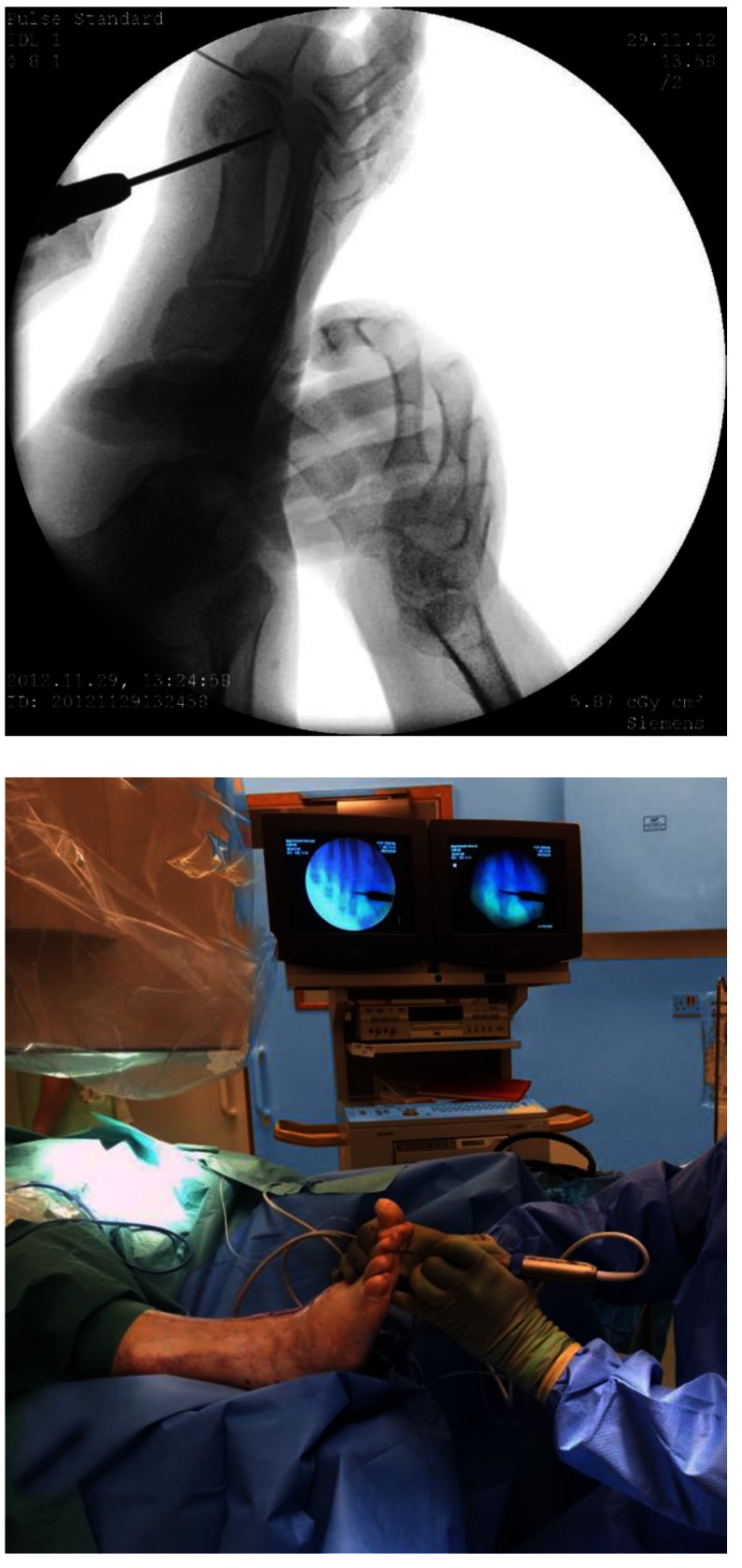
Distal metatarsal osteotomy under fluoroscopic guidance

For the MP extension and subluxation, the GRECMIP [**9**] surgical formula is the following: DMMO + EDL/ EDB tenotomy more proximal than the MP level, +/-MP arthrolysis (DMMO = distal metatarsal minimally osteotomy, EDL = extensor digitorum longus, EDB = extensor digitorum brevis, MP = metatarsophalangeal joint). The performance of CONVERSE DMMO, which is a more vertical and proximal distal metatarsal minimally osteotomy, having two principles, is indicated in the diabetic foot, as in **Fig. 5**: positions the heads up and shortens the least possible metatarsals.

**Equinus deformity - posterior crural dominance**

**Fig. 6:**
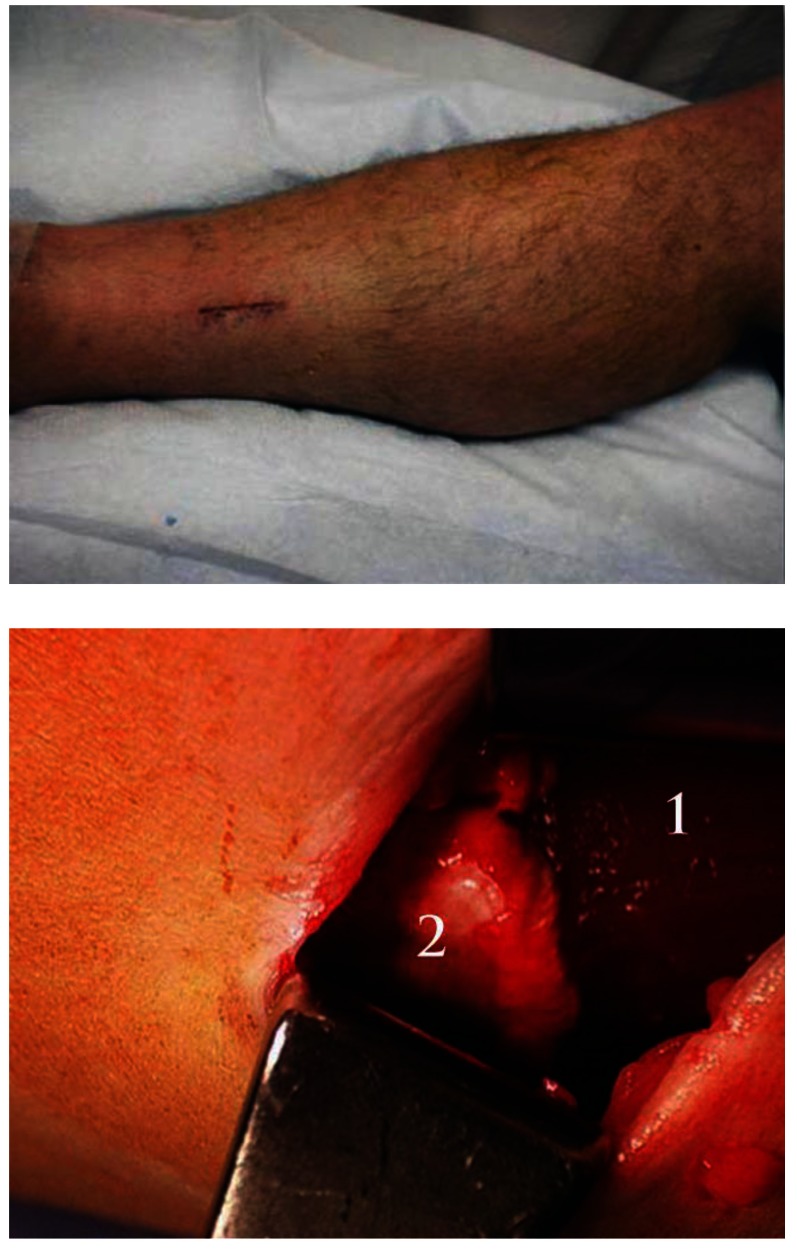
Gastrocnemius release (incision on the left, intraoperative aspect with 1. gastrocnemius muscle and 2. Released fascia on the right)

Equinus is defined as an insufficient ankle joint dorsiflexion for normal gait, resulting in lower extremity pathology. The equinus with an existing dorsiflexion of less than 5 degrees is associated with important gait compensation and increased forefoot pressures, determining a greater incidence of the foot pathology [**10**]. The surgical management of the equinus deformity consists in a gastrocnemius release or a percutaneous Achilles tendon lengthening. The incidence of failure of foot surgeries from the point of the deformity correction to the point of pain reduction is significantly less when gastrocnemius lengthening is performed (NB: our technique uses the gastrocnemius intramuscular aponeurotic recession). The procedure involves a single transection of only the gastrocnemius aponeurosis, supporting the gastrocnemius muscle belly through a limited (3cm), open, medial approach in the calf (**Fig. 6**).

**Charcot deformity**

The rocker bottom deformity is a midfoot collapse resulting from the Charcot related osseous destruction. The realignment and fusion by a midfoot osteotomy, correction to a plantigrade foot, and as rigid as possible internal fixation are solutions to this problem. The midfoot osteotomies often avoid the extensive soft tissue exposure required for multiple joint arthrodesis procedures, because these procedures can be performed through minimum or percutaneous incisions. Reduction is performed by wedge osteotomy and bone removal. The internal fixation must be done with an intramedullary screw (at least two for both columns – from talus to first metatarsal and from calcaneus to the fourth metatarsal) or multiple percutaneous screw (preferred 3,5 - 4 mm HCS headless compression-type screws) for midfoot-tarsometatarsal joints.

**Fig. 7:**
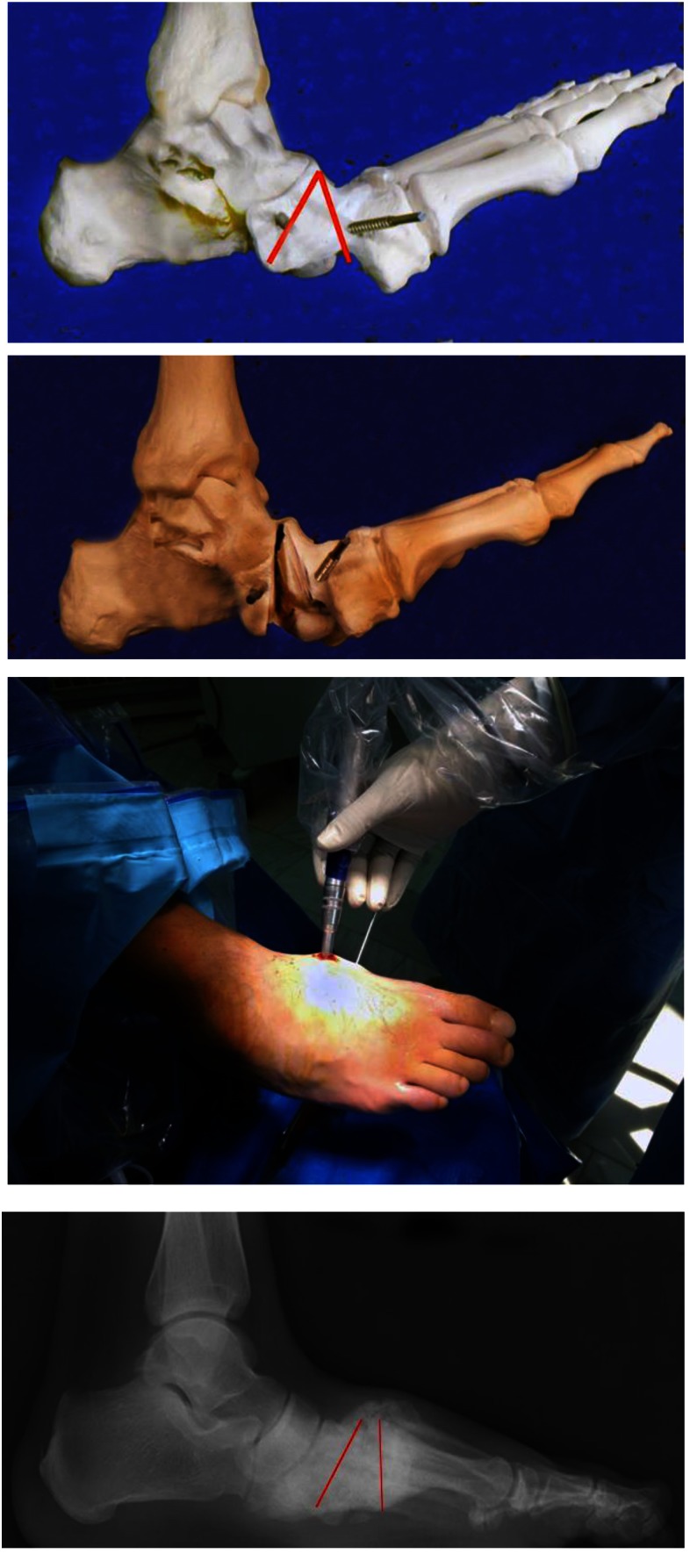
Percutaneous osteotomy – surgical planning using 3D printed models; intraoperative aspect with guiding K-wires and high-speed burr under fluoroscopic guidance

The percutaneous technique (as endorsed by GRECMIP – **Fig. 7**) involves midfoot arthrodesis through small incisions – with percutaneous bone shaving with a burr along with two guiding K-wires inserted at angles and positions established by the preoperative planning for the midfoot wedge osteotomy. The bone, which was shaved, remains as a bone graft.

**Fig. 8:**
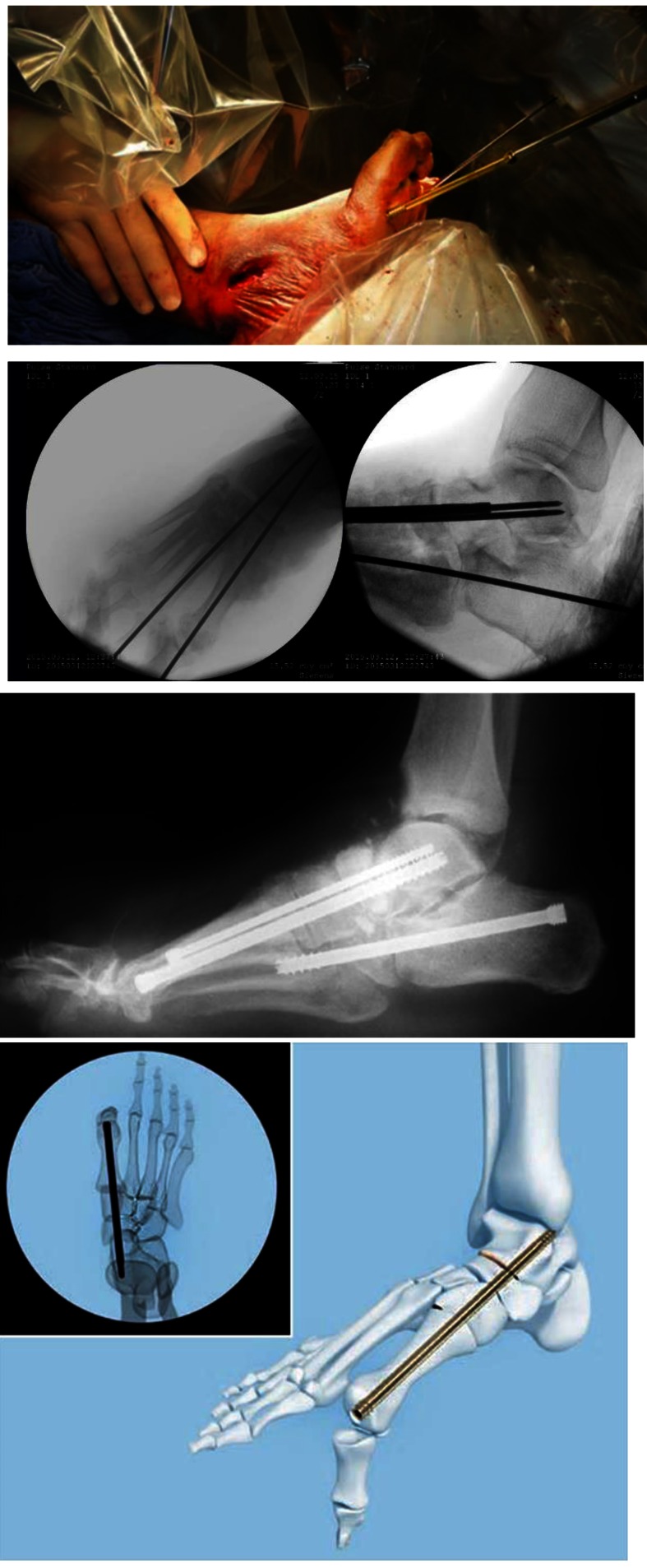
Midfoot Fusion Bolt technique – minimal incision for arthrodesis followed by the insertion of 6,5 mm screws; X-ray postoperative aspect of the foot and surgical illustration (MFB® Synthes DePuy)

When medial, lateral and subtalar fusion is performed, this can be done through a limited open approach and intramedullary screws (6,5 mm solid screw – MFB Mid Fusion Bolts) that can be placed either percutaneously or in a minimally invasive manner. The position of the guide wire, the confirmation of the reduction (correct and realigned deformities) and the screws insertion is assessed with the fluoroscopic image intensifier. In addition, this screw is inserted in such a way that the risk of hardware exposure and soft tissue healing complications is minimized (**Fig. 8**).

Another frequently applied technique involves the percutaneous bone shaving as a minimally invasive approach that can remove the plantar osseous prominence through a small incision that is away from the plantar weight-bearing area. The main indication is related to the advanced age or the altered general status of the patient, but also requires a stable foot, or in the case of previous multiple flap surgeries, when the blood vessel network may have been altered. The location of the incision at the side of the foot has the advantage of a less potential of contamination of the bone, not disturbing the vascular supply to the foot, keeping the incision off of the plantar surface of the foot avoids painful scarring at the weight-bearing area, and risk of creating another high pressure zone subject to further ulcer formation [**11**]. Moreover, it is easier to remove the bone prominence and create a flat surface with the burr inserted at the side of the foot. It is a useful surgical technique, which modifies the remaining plantar pressure points after the correction of the foot deformity. The plantar bone shaving will not affect the midfoot stability because it is already stabilized by the internal implant. 

## Conclusions 

The first objective of any surgical intervention on the diabetic foot is to correctly diagnose the stage and location of the complication. Several factors to consider when the surgical intervention is indicated are the following [**12**]: the vascular supply to the affected limb, the anatomic location of collapse, the patient’s co-morbidities and medical stability, the patient’s ability to comply with postoperative weight-bearing restrictions. With such complex clinical situations, it is important to reduce the extent of the surgery while amplifying the mechanical resistance of the constructs. Many of the minimally invasive techniques answer to these requirements.

The goal of a good corrective foot surgery is to re-establish a plantigrade – (stable, realigned) foot during stance, which implies that the first metatarsal head, fifth metatarsal head, and calcaneus are on the same plane during stance. The classical midfoot osteotomy techniques require the removal of the bone altered in excess, the reduction of the dislocated bones, and the stabilization with internal fixation (screw fixation or plantar plating). These invasive surgical procedures typically resulted in a non-anatomic correction (e.g., shortening of the foot or incomplete deformity correction), and occasionally neurovascular complications, incision healing problems, infection, etc. In order to avoid these complications, minimally invasive techniques have been developed and successfully included in modern therapeutic protocols. 
